# Designing and fabrication of electrochemical nano-biosensor for the fast detection of SARS-CoV-2-RNA

**DOI:** 10.1038/s41598-023-32168-5

**Published:** 2023-03-29

**Authors:** Heba A. Hussein, Amro Hanora, Samar M. Solyman, Rabeay Y. A. Hassan

**Affiliations:** 1 Virology Department, Animal Health Research Institute (AHRI), Agricultural Research Center (ARC), Giza, 12619 Egypt; 2 Department of Microbiology and Immunology, College of Pharmacy, Suez Canal University, Ismailia, Egypt; 3 Biosensors Research Laboratory, Zewail City of Science and Technology, 6Th October City, Giza, 12578 Egypt

**Keywords:** Health care, Chemistry

## Abstract

SARS-CoV-2 caused a global panic among populations. Rapid diagnostic procedures for the virus are crucial for disease control. Thus, the designed signature probe from a highly conserved region of the virus was chemically immobilized onto the nanostructured-AuNPs/WO_3_-screen printed electrodes. Different concentrations of the matched oligonucleotides were spiked to test the specificity of the hybridization affinity whereas the electrochemical impedance spectroscopy was used for tracking the electrochemical performance. After a full assay optimization, limits of detection and quantification were calculated based on linear regression and were valued at 298 and 994 fM, respectively. Further, the high performance of the fabricated RNA-sensor chips was confirmed after testing the interference status in the presence of the mismatched oligos in one nucleotide and completely one. Worthy to mention that the single-stranded matched oligos can be hybridized to the immobilized probe in 5 min at room temperature. The designed disposable sensor chips are capable of detecting the virus genome directly. Therefore, the chips are a rapid tool for SARS-CoV-2 detection.

## Introduction

Since December 2019, the globe was stricken by a life-threatening respiratory disease caused by the severe acute respiratory syndrome Coronavirus-2 (SARS-CoV-2). The disease is first disclosed in Wuhan, China by severe respiratory manifestations^1,2^. The virus is a non-segmented, positive sense, enveloped RNA virus that belongs to the genus *Betacoronavirus* of the family *Coronaviridae*^3^. The genome of SARS-CoV-2 is 29.9 Kb having 38% of G/C pairs^4^. The virus has high mutation rates that are clearly reflected in the pathogenicity, infectivity, virus transmission, and spread as well^5^. The viral genomic codes encrypt 27 proteins including 16 non-structural proteins (NSP) and other structural proteins^6^. Its spike protein (S-protein) plays an essential role in the virus binding to the host cell. Mediating by the specific cellular proteases, the S-protein is cleaved into S1 and S2 subunits, whereas, cleavage is essential for the virus entry and integration into the host cell^7^. The genomic thread is organized as 5′ UTR ORF 1a/b-S (Spike)-E (envelope)-M (Membrane)-N (Nucleocapsid)-3′ UTR poly (A) tail, the accessory proteins are interspersed within the structural proteins. SARS-CoV-2 has about 80% and 96% nucleotides similar to SARS-CoV and bat-CoV-RaTG13, respectively^8–10^.

Biosensors are outstanding investigation tools used for the fast detection of infectious microorganisms, especially viruses^11^. Recently, most of the scientific research shed light on the COVID-19 crisis. Thus, various studies were carried out for the selective and prompt detection of SARS-CoV-2 using different types of biosensing technologies^12,13^. For instance, a fabricated biosensor based on an entropy-driven amplified electrochemiluminescence which conducted to link the Ru(bpy)_3_^2+^ modified S3 to the linear ssDNA at the vertex of the DNA tetrahedron which was used for detection of SARS-CoV-2-RNA-dependant RNA polymerase gene (RdRp). A DNA tetrahedral structure reduced the cross-reactivity and increased the sensitivity of the ssDNA by enhancing the signal intensity of the fabricated sensor. The limit of detection was 2.67 fM which provided a reliable platform for clinical analysis^14^. Moreover, an ultrasensitive colorimetric/luminescence-developed biosensor was used to detect SARS-CoV-2 RNA and its variants in the patient's samples using the PHAsed NASBA-Translation Optical Method (PHANTOM). Isothermal amplification of the viral genome which is coupled with the fabricated biosensor resulted in the conformational switch to translate a nano-lantern (LacZ), a reporter protein which resulted in a color/luminescence formation that is easily quantified by spectrophotometry and visualized by the naked eye. The designed biosensor showed the ability to detect the viral genome as low as 100 copies^15^.

Based on, the catalytic and optical properties of gold nanoparticles (AuNPs), the particles are intensively used in various diagnostic techniques. For instance, AuNPs-coated with thiolated antisense oligonucleotide-capped probes were implemented to detect SARS-CoV-2 -N-protein. The probe was immobilized on a paper-based electrochemical platform. The calculated limit of detection (LOD) of the plasmonic biosensor was 0.18 ng/µL of virus-N protein in clinical samples and the virus culture within 5 min at 6.9 copies/µL^16^. In addition, another thiol-modified antisense N-oligonucleotides (ASOs) capped with AuNPs-based biosensor was further developed. The biosensor provided a naked-eye detection by the color shifting from violet to dark-blue color due to the hybridization of the extracted SARS-CoV-2-RNA to the immobilized oligos to form Au-Aso-RNA agglomerate. The addition of the RNaseH cleaved the RNA strand from the RNA–DNA hybrid leading to a visible detected precipitate that is mediated by the plasmonic signal response of additional agglomeration of the AuNPs. The whole sensing time needed for the detection and signal processing of the viral RNA extraction was 10 min while the LOD was 0.18 ng/µL^17^.

Furthermore, a dual plasmonic biosensor based on the plasmonic photothermal (PPT) effect and localized surface plasmon resonance (LSPR) response was introduced. Functionalized two-dimensional gold nanoislands (AuNIs) with complementary DNA oligos were fabricated for the selective detection of the hybridized SARS-CoV-2 nucleic acid. The plasmonic resonance frequency was investigated after the thermoplasmonic heat was generated to the AuNIs chips while PPT is oriented to elevate the in-situ hybridization temperature that enhanced the discrimination of the virus gene over the others. The estimated LOD of the dual biosensor was 0.22 pM and provided a target platform for the SARS-CoV-2 gene in the mixture of the multi-genes^18^.

For variant detection, an electrochemical CRISPR-Cas-12a-based biosensor was fabricated using the SARS-CoV-2 Delta Spike gene sequence on the base of the electro-deposited gold. Within 1 h, the biosensor was applied to detect the virus nucleic acid at a limit of 50 fM without genome amplification^19^. Recently, ultrasensitive and rapid electromechanical detection of the SARS-CoV-2 RNA in nasopharyngeal specimens without the need for viral RNA extraction and amplification. A proposed immobilized molecular system of aptamer probes bound with a single-stranded DNA cantilever fixed by self-assembled tetrahedral double-structured DNA. The system is loaded on a liquid-gated graphene field-effect transistor. With a detection limit estimated from one to two copies in 100 µL, clinical specimens from 33 patients with COVID-19, recorded threshold values of 24.9–41.3, and 54 negative controls were applied on the fabricated biosensors and the signals were investigated within four minutes^20^. Furthermore, a self-actuated electrochemical biosensor was modified to detect a few copies of un-amplified SARS-CoV-2 RNA in samples. The system consisted of a tentacle and trunk immobilized on a graphene-based electrode, and the probe and the electrochemical labels were bound to the tentacle with the upright orientation. Once the nucleic acid is identified, the tentacle/label is subsequently actuated downward and produced the electrochemical remarkable response recorded by the square wave voltammetry. Using this developed assay, the viral genome was determined within 1 min and the detection limit was estimated at 4 copies in 80 µL^21^.

Worthy, nanomaterials have been extensively used in the fabrication of electrochemical nano-biosensors to enable high electro-catalytic activity, and electrical conductivity, and to support the orientation and stability of the bio-recognition element(s). Thus, in this study, the designing and fabrication of nano-SARS-CoV-2-RNA biosensor chips were achieved for the rapid detection of the virus RNA based on the immobilized conserved oligonucleotide sequence for the selective identification of the virus genome using disposable sensor chips.

## Material and methods

### Detection of the most conserved sequences in the SARS-CoV-2 genome

Reference genomes (92) of SARS-CoV-2 were downloaded from NCBI (https://www.ncbi.nlm.nih.gov/genome/browse/#!/viruses/86693). Clustal X was downloaded and installed to perform multiple sequence alignment (http://www.clustal.org/download/current) for the detection of the conserved regions in the virus genome. BLASTn tool was used to align SARS-CoV-2 conserved sequences against human (taxid: 9605), bacteria (taxid: 2) and viruses (taxid: 10239) with the exclusion of SARS-CoV-2 (taxid: 2697049). Signature probes, matched oligonucleotides, Mismatched in one nucleotide, and completely mismatched oligonucleotides were then designed and sent for synthesis (Introgen co., USA).

### Modification of the biosensor surface with nanomaterials

The biosensor surface was modified with metal oxide nanomaterials combined with gold nanoparticles (AuNPs). The used metal oxides included vanadium oxide (V_3_O_5_), antimony oxide (Sb_2_O_3_), selenium oxide (SeO_2_), zirconium oxide (ZrO_2_), cerium oxide (CeO_2_), manganese dioxide (MnO_2_), Lanthanum oxide (La_2_O_3_), titanium oxide (TiO_2_), tungsten-oxide (WO_3_), and germanium oxide (GeO_2_). The electrochemical responses of the modified electrodes were investigated in ferricyanide as the standard redox probe using cyclic voltammetry (CV) at the potential ranging from − 0.4 to 1.0 V and the applied scan rate of 50 mV/s. Moreover, electrochemical impedimetric spectroscopy (EIS) was applied at a direct current (DC) of 0.25 V and the frequency ranged from 0.1 to 10,000 Hz.

### Cross-linker candidates for the immobilization of SARS-CoV-2 oligonucleotides

For the immobilization of SARS-CoV-2 selected oligos, different cross-linkers were tested for the effective immobilization of the selective bio-recognition elements (the extracted virus RNA) on the electrode surface. Therefore, 4-amino thiophenol (4-ATP) (Sigma-Aldrich, USA), EDC/NHS coupling by 1-ethyl-3(3-dimethyl aminopropyl) carbodiimide hydrochloride (EDC) and *N*-Hydroxysulfosuccinimide sodium salts (NHS), (Sigma-Aldrich, USA). A mixture of 0.2 M EDC and 0.05 M NHS was used for the functionalization of the AuNPs/WO_3_-modified electrode for 30 min at room temperature. For the activation of the electrode surface, diphenylamine (DPA, ACS reagent ≥ 99%, Sigma-Aldrich), 3.0 mM in ethanolic solution was used for 45 min. Additionally, the modified electrodes were functionalized using 3 mM of ethanolic solution of 4-ATP for 16 h at 4 °C to form a self-assembled monolayer (SAM)^22,23^. Afterward, the electrodes were rinsed with ethanol to remove excess non-reactive layers of ATP.

### Immobilization of the SARS-CoV-2 Probe on the functionalized biosensor

The probe was used at different concentrations (1, 10, 25, 50, and 100 µM), and the significance, as well as the response of the electrodes, was tested in the presence of the hybridized matched oligos (10 µM) by impedimetric spectroscopy. A 25 µM of the designed probe (suspended in 20 mM of tris buffer (TE), pH = 8) was immobilized onto the ATP-functionalized biosensor chips. The SARS-CoV-2 signature (i.e. probe, the sequence as given in Table 1) was self-assembled on the electrode surface for 2 h at room temperature. Afterward, the electrodes were washed three times with PBS and TE to remove the non-immobilized oligos.

**Table 1:** Sequences of the designed oligos used for the construction of the SARS-CoV-2 RNA biosensor.

No	Item	Designed sequence*
1	Probe (signature)	5′ GTA TAA TTA ATA ACT AAT TAC TGT 3′
2	Matched oligos	5′ ACA GTA ATT AGT TAT TAA TTA TAC 3′
3	Mismatched in one nucleotide	5′ ACA GTA ATT AGA TAT TAA TTA TAC 3′
4	Completely mismatched	5′ CGA AAG GAC GTC TTG AAC ATG CC 3′

*The designed primers were provided by Introgen Co., USA. The mismatched oligo in one nucleotide (Thiamine nucleotide of the target Oligo replaced by Adenine in the mismatched Oligo).

The blocking step is necessitated for the inactivation of the pinholes’ non-reactive sites of the cross-linker. Thus, the electrodes were immersed in 10 mM of 6-Mercapto1-Hexanol, 97% (Sigma-Aldrich, USA) for 15 min at 25 °C.

Different DC values (ranging from 0.0 to 1.2 V) were applied to the electrode before and after the hybridization (i.e. Probe and after hybridization with the matched oligos). The experiment was applied to the prepared RNA-based biosensor chips in 3.0 mM of the redox mediator (FCN) in TE.

### Selectivity testing of the SARS-CoV-2 RNA biosensor

The selectivity performance of the fabricated sensor chips was tested towards foreign oligos (non-targeting). In this regard, the functionalized electrodes were tested in the presence of one nucleotide-mismatched, and completely mismatched oligos in comparison to the target oligo (Table 1). The chips were loaded with 10 µL of 10 µM of the tested oligos for 5 min at room temperature. Afterward, the prepared RNA biosensors were washed three times with TE buffer and subjected to electrochemical investigations.

### The hybridization test of RNA-based electrodes

The fabricated RNA biosensors were tested for their selective hybridization in the presence of the designed matched oligos (Table 1). Different concentrations of the matched oligos ranged from 10 fM to 1.0 µM. The prepared biosensors were electrochemically investigated in FCN/PBS using EIS at 0.25 V DC. The primer suspensions were loaded on the sensor´s surface for 5 min, followed by washing with PBS and TE to remove any excess non-reactants.

### Statistical and data analysis

All obtained electrochemical results are presented as average ± SD from at least three individual successive experiments using the fabricated RNA- sensor’s chips. Statistical significance was determined by statistical hypothesis testing where the significance of the values was assumed to be *p* < 0.05. Based on the obtained calibration curve, the limit of detection (LOD) and quantification (LOQ) were calculated. All the statistical analyses as well as drawing of all figures were performed using Origin-Lab software.

## Results and discussion

### Conserved regions on SARS-CoV-2 genome and signature probe design

The following sequences were found to be conserved in the used reference genomes of *SARS-CoV-2* (GTATAATTAATAACTAATTACTGTCGTTGACAGGACACGAGTAACTCGTCT), length: 51 nucleotides and, (AATCAAGACTATTCAACCAAGGGTTGAAAAGAAAAAGCTTGATGGCTTTATGGGTAGAATTCG A), length: 64 nucleotides.

The two conserved sequences were used as query to perform BLASTn sequence alignments and the result showed sequence alignment against human and bacteria sequences showed no significant similarity, however, BLASTn sequence alignment against viruses (with the exclusion of SARS-CoV-2) was matched only with Bat coronavirus which is an acceptable result due to the evolution of SARS-CoV-2 from bat virus.

### Nanomaterials selection for the SARS-CoV-2 RNA biosensor

**Figure 1:**
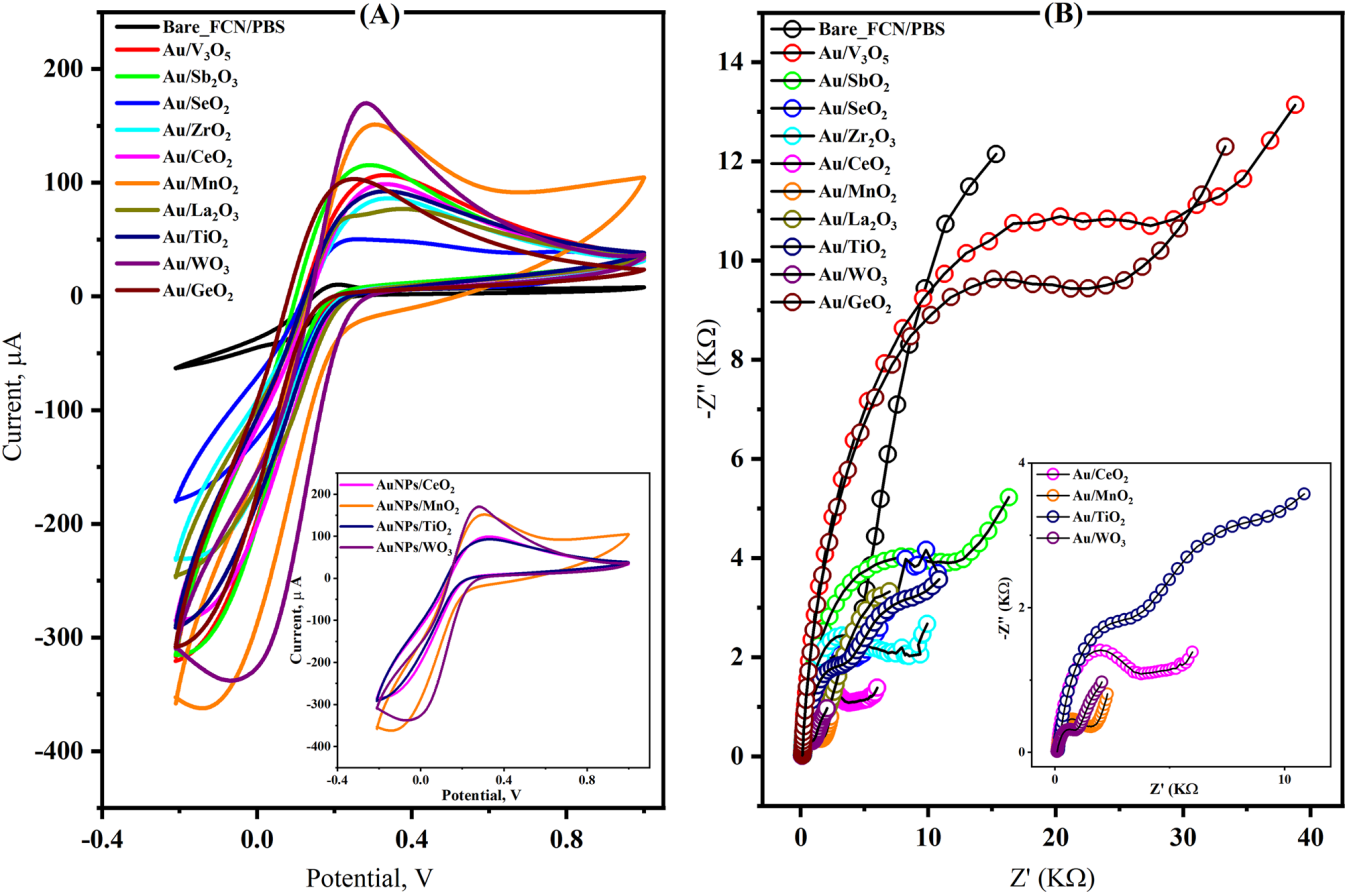
The electrochemical investigations of the AuNPs/metal oxides modified SPEs. The AuNPs/WO_3_ showed the highest significant response for electron transfer in FCN/PBS (**A**), and a lower impedimetric response among the other nanomaterials (**B**).

Sensor surface modification with nanomaterials was conducted for enhancing the performance of the newly developed RNA biosensor. To select the best nanomaterial, electrochemical experiments (CV and EIS) were carried out on screen-printed electrodes (SPEs) modified with gold nanoparticles solely or combined with one of the following metal oxides (V_3_O_5_, Sb_2_O_3_, SeO_2_, ZrO_2_, CeO_2_, La_2_O_3_, TiO_2_, WO_3_, or GeO_2_). As a result, higher voltammetric signals combined with lower charger transfer resistances were obtained from all nanomaterials-based SPEs. However, the nanocomposites (metal/metal oxides) increased the sensor surface capacity and improved the sensor conductivity and electrocatalytic properties. Among the long-tested list of nanomaterials, AuNPs/WO_3_ nanocomposite demonstrates the highest redox reaction with the lowest charge transfer resistance (Fig. 1). Thus, the nanocomposite (AuNPs/WO_3_) was selected as a platform for the fabrication of the SARS-CoV-2-RNA biosensor.

### Fabrication of SARS-CoV-2 RNA biosensor

A self-assembled monolayer (SAM) of 4-amino thiophenol (4-ATP) was formed on the AuNPs/WO_3_-modified electrodes overnight incubated with 3.0 mM of ATP. The thiol groups of the formed layer were conjugated with the gold nanoparticles loaded on the electrode surface.

**Figure 2:**
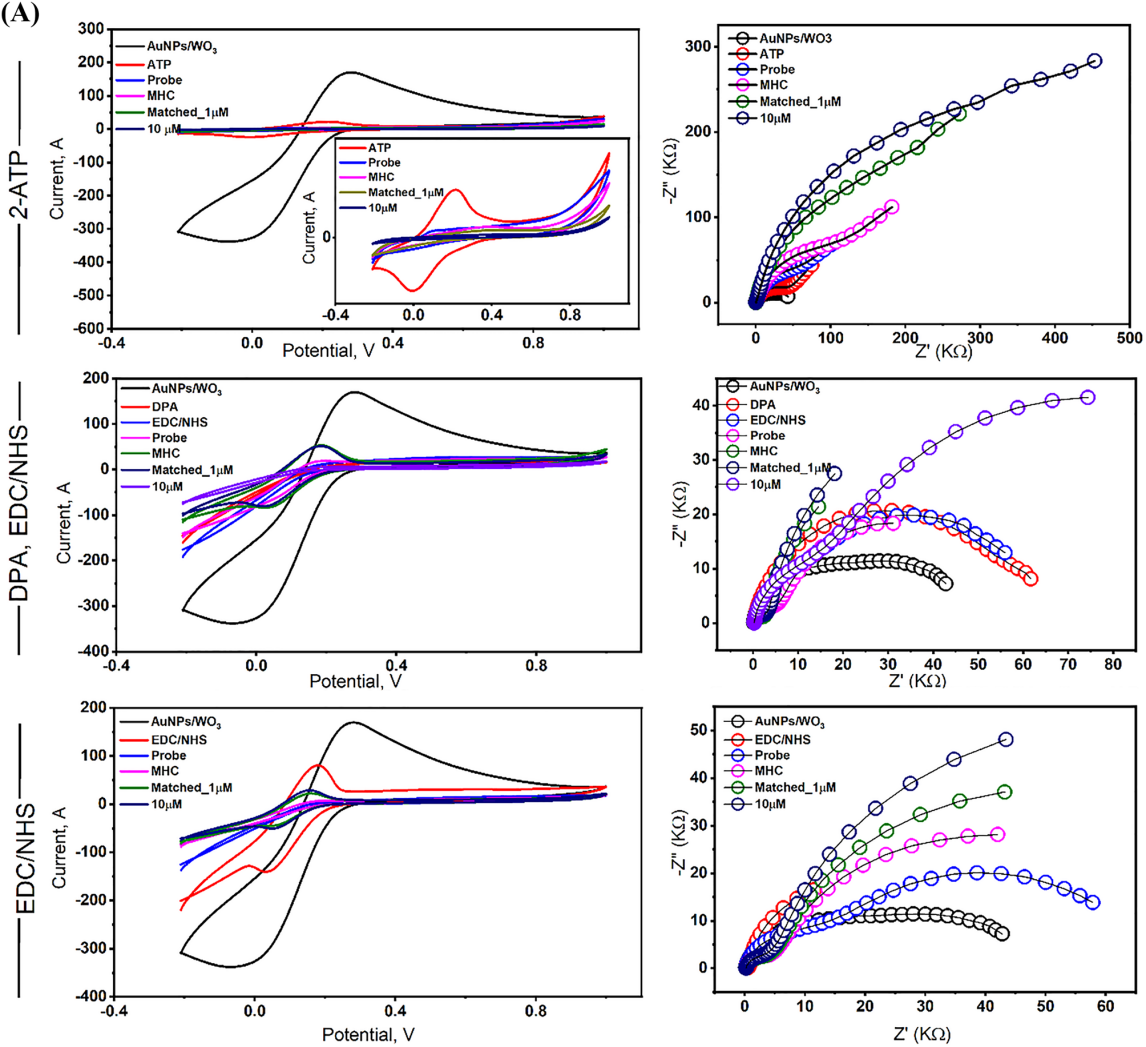

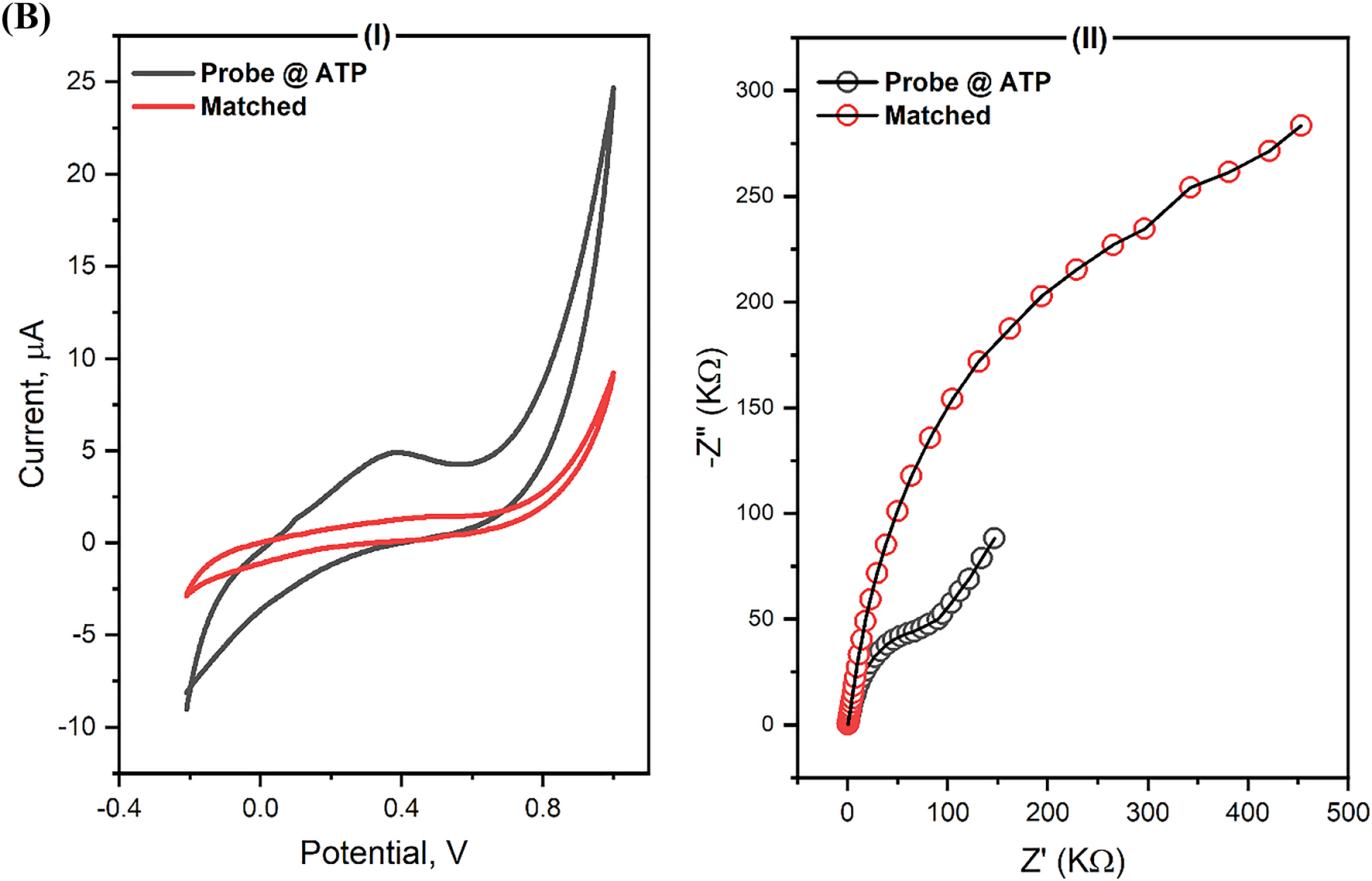
(**A**) Using different crosslinking agents including 4-ATP, EDC/NHS, and DPA/EDC/NHS. The voltammetric and the impedimetric signals were investigated in FCN/TE. Modified surfaces with the 4*-*ATP showed the highest significant response. (**B**) The *4-ATP*-based electrode showed a remarkably decrease in the voltammetric signals after the hybridization with the matched oligonucleotides (I). Significant impedimetric signals were obtained for the hybridized attached probe (II).

Formerly, different cross-linkers were tested for their activity on the electrode surface. For instance, EDC/NHS coupling agents with and without DPA surface activation, and *4-*ATP (Fig. 2A). The electrochemical responses of the fabricated electrodes towards the matched SARS-CoV-2 oligos (1.0 and 10 µM) were investigated in FCN/TE solution. The best signals were recorded with the ATP-based RNA sensor rather than the EDC/NHS coupling by around 80 times impedimetric response. Strong inhibition in the voltammetric signals was obtained after the hybridization and subsequently a significant impedimetric response in case of the ATP-based sensor, which contributed to the sensor's surface covering and strong binding to the lining probe as given in Fig. 2B. Thus, the 4-ATP was chosen for the proper covalent immobilization of the SARS-CoV-2 RNA.

### Immobilization of SARS-CoV-2 probe on the biosensor chips

**Figure 3:**
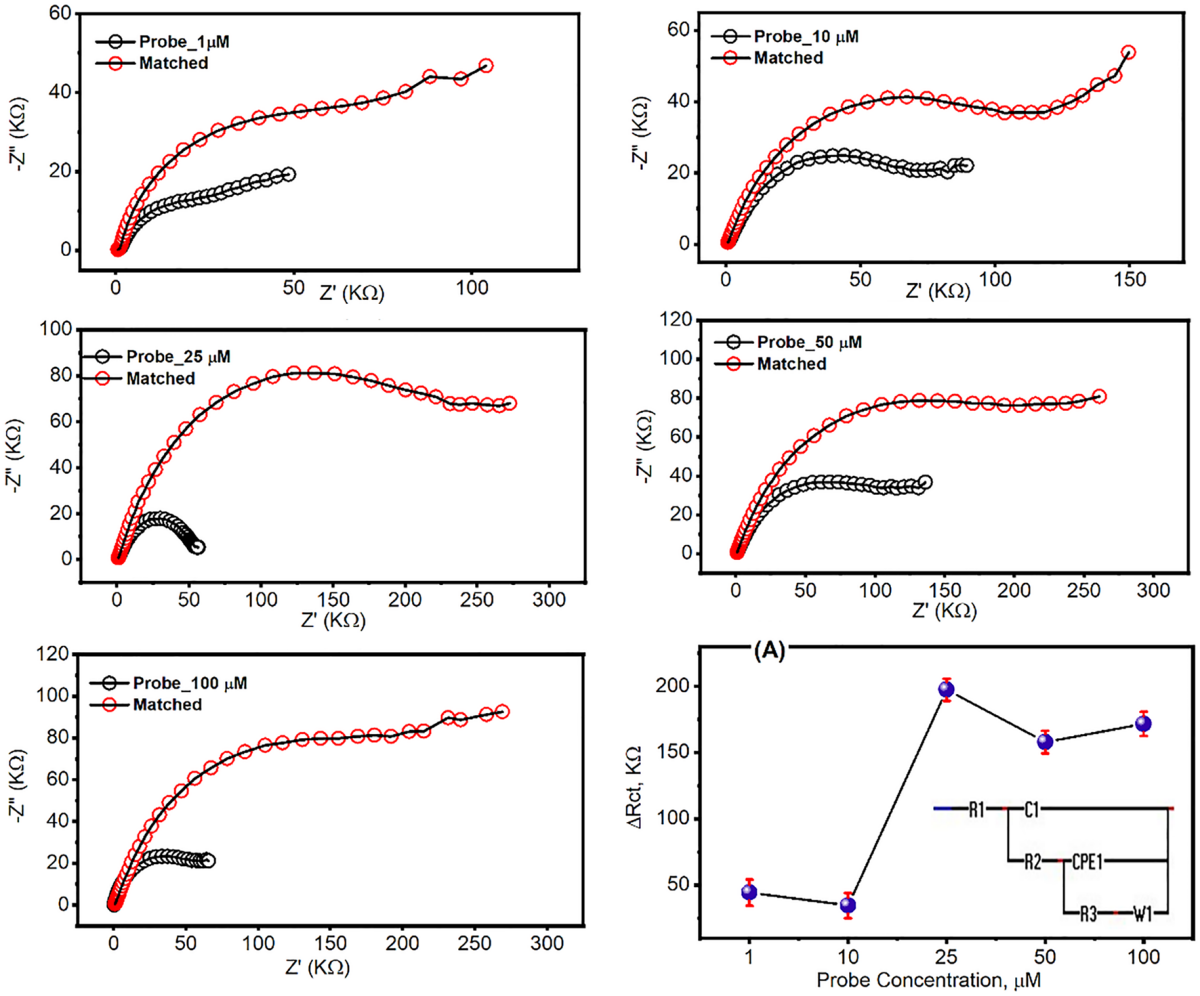
Different concentrations of SARS-CoV-2 probe immobilized on the electrode's surface. The use of 25 µM exhibited the highest EIS signals representing the most effective selective binding. Accordingly, the concentration was assigned for the fabrication of the SARS-CoV-2 RNA biosensor.

The SARS-CoV-2 signature (probe) was immobilized on the electrode surface by incubation for 2 h at room temperature. The oligos were attached to 4*-*ATP on the electrode surface. A wide range of probe concentrations (1.0 to 100 µM) was used and the impedimetric signals were collected for each concentration hybridized with 10 µM of the matched oligos. The best signal was investigated in case of 25 µM of the probe as shown in Fig. 3.

**Figure 4:**
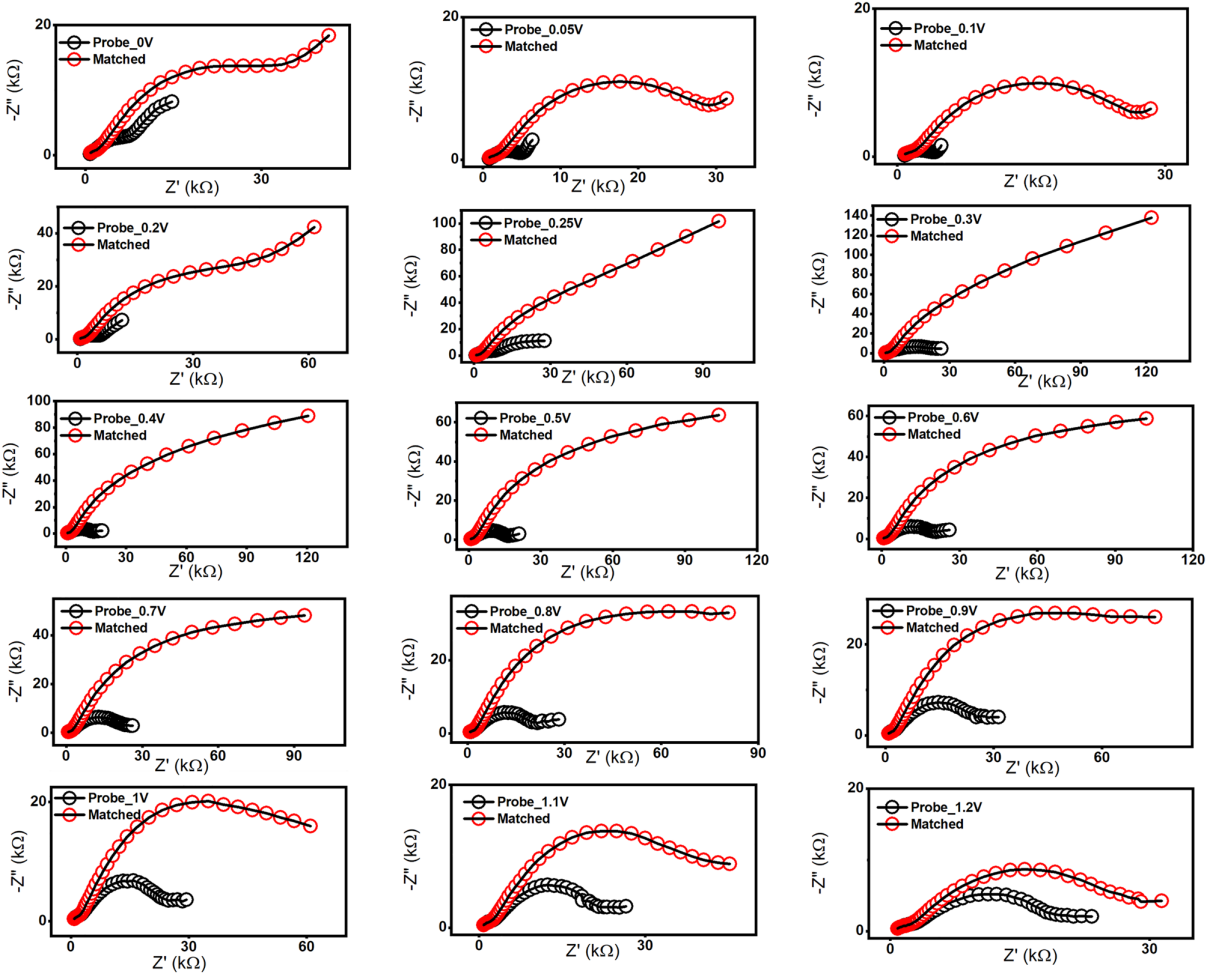
Effect of applied DC on the fabricated biosensor chips. Remarkable signals were obtained at 0.3 V. The study was applied before and after the hybridization with 10 µM of the matched oligonucleotide.

Moreover, the effect of the applied direct current (DC) range was studied from 0.0 to 1.2 V. In this regard, the EIS signal of the RNA-biosensor was recorded at each variable DC value. As a result, the best biosensing performance was obtained at 0.3 V (Fig. 4).

Consequently, the hybridization time of the matched oligos (target) bound to the signature probe was tested over different time points (5, 10, and 15 min) at room temperature. A sensing time of 5 min was enough for the effective binding of the viral RNA as given in Supplementary Data Figure S1.

### Hybridization of the immobilized oligos with the matched target

**Figure 5:**
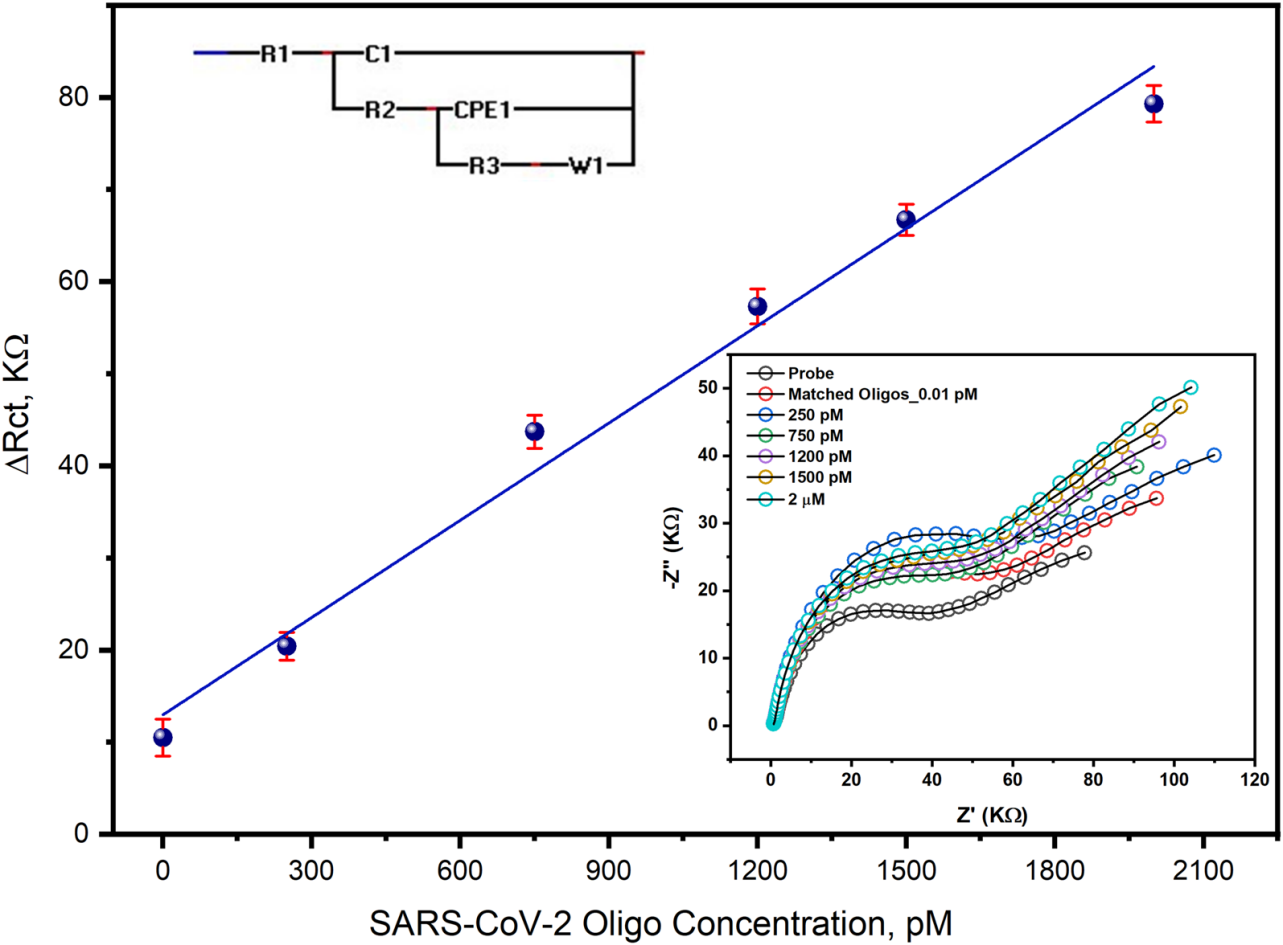
Standard calibration curve of the SARS-CoV-2 RNA biosensor using different concentrations of the virus signature nucleotide sequence ranging from 10 fM to 2 µM. In FCN mediating Tris buffer, the impedimetric signals were investigated before and after the hybridization with the different concentrations of the matched oligos for three individual readings. ΔR_ct_ values determined after fitting the resulted signals using the Randles cell circuit where R1: FCN/TE buffer resistance, C1: capacitance of sensor surface, R2: resistance of immobilized probe, CPE1: constant phase elements of polymerized amino thiophenol monolayer, R3: the resistance of the hybridized matched oligo, and W1: Warburg impedance for the FCN diffusion on the electrode surface. The LOD and LOQ were 298.1 and 993.7 fM, respectively. The obtained R2 from the linear regression was 0.99 and *P*-value was < 0.0001.

To test the performance of the fabricated RNA biosensor, a different wide range of the matched oligos concentrations (i.e. 0.01, 250, 750, 1200, 1500, and 2000 picoMolar). The impedimetric signals were investigated before and after the hybridization of the matched oligos. According to the resulting difference in the charge transfer resistance (ΔR_ct_), the standard calibration curve was constructed from the EIS response of each matched oligos concentration as shown in Fig. 5. The limits of detection (LOD) and quantification (LOQ) were estimated and their resulting values were 298 and 994 fM, respectively, which indicated the high sensitivity of the fabricated biosensor chips more than the other proposed biosensors based on thiol-modified antisense N-oligonucleotides (ASOs) capped with gold nanoparticles (AuNPs) which provided a sensitivity of 0.18 ng/µL^16,17^. Interestingly, the newly designed chips in this study provided a high sensitivity, selectivity, and fast detection of the targeting probe within 5 min.

### Testing the fabricated chips in the presence of the mismatched oligos

**Figure 6:**
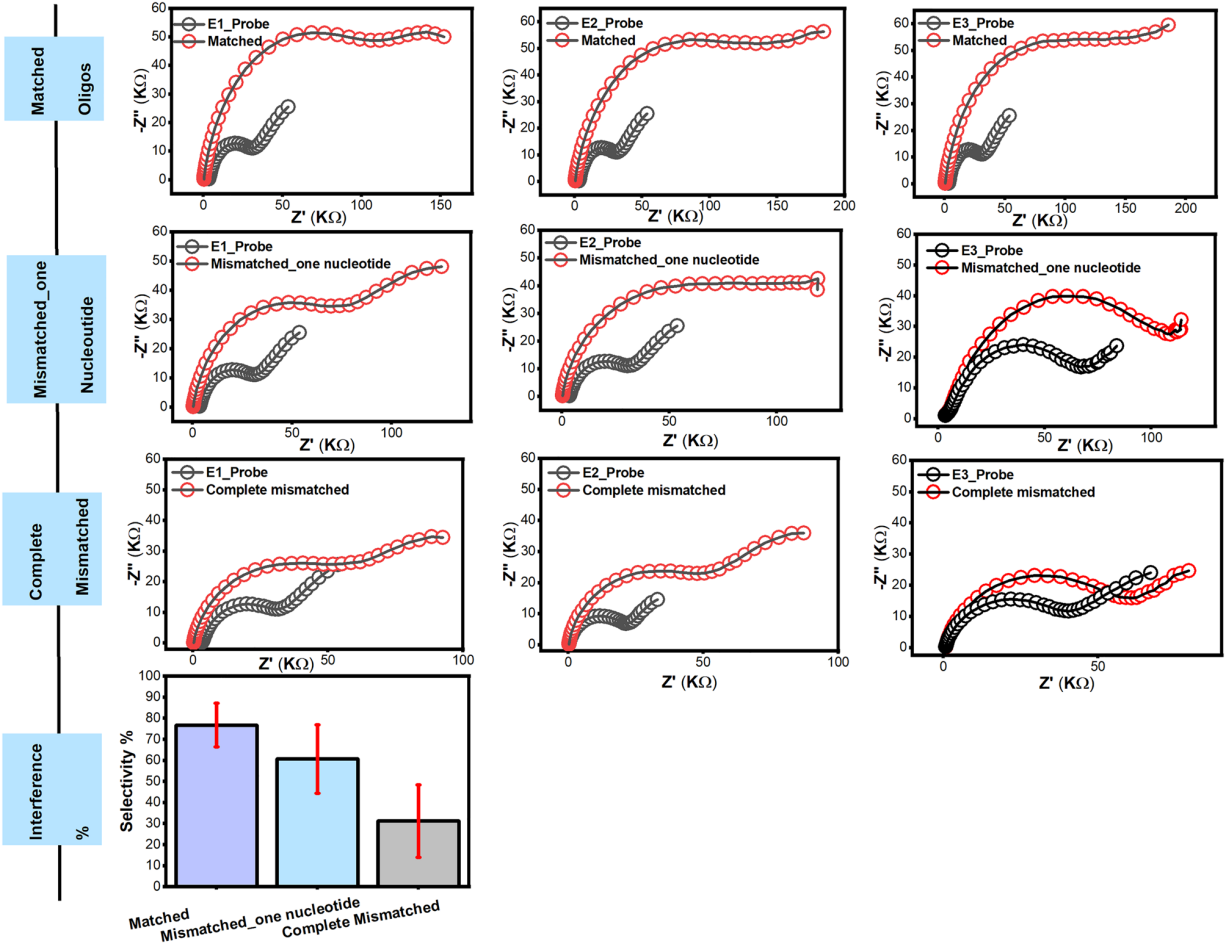
The impedimetric response of the fabricated SARS-CoV-2-RNA biosensor chips towards the mismatched designed Oligos (one nucleotide, completely mismatched), the chips were used in triplicates. The ΔRct was estimated and the percentage of the interference was obtained. The electrodes showed a response of 79% and 40.6%, respectively. The error bars expressed the standard deviations (SDs) between the three electrode readings.

For testing the performance of the SARS-CoV-2 biosensor, the chips were further tested for their selectivity in the presence of other mismatched oligonucleotides (i.e. mismatched in one nucleotide and completely mismatched oligos) as interfering reactant oligos. The biosensor showed a selective hybridization towards the target-matched oligos and showed a slight interfering response towards the mismatched oligos in one nucleotide by 79% responsive electrochemical action. This might be attributed to the mismatching with the target oligos in one nucleotide while the biosensor showed an unremarkable response towards the complete mismatched oligos by 40.6% as shown in Fig. 6.

### The life span of the fabricated biosensor chips

The fabricated chips were stable after 2 weeks of their construction as the investigated electrochemical signals have no remarkable changes. Afterward, a 5–10% decrease in the obtained signals towards the matched oligonucleotides. The chips remain stable for 1–2 months when it kept completely dried at 4 °C. The chips were rinsed with TE buffer before use and subjected to three successive voltammetric activations before use in TE/FCN.

## Conclusion

The fabricated biosensor is based on the immobilization of a designed probe of the most conversed oligonucleotides in the Nucleoprotein gene of SARS-CoV-2. The designed oligos are representative of a signature for virus detection. To enhance the electrochemical biosensing performance, a nanocomposite of AuNPs/WO_3_ was selected as a platform for the RNA-sensor construction. The modified sensors' surface with the gold nanoparticles enabled the thiolation with the lining layer of the ATP for probe immobilization. A 25 µM of probe was used to functionalize the RNA biosensor. A wide range of the matched oligos concentration (10 fM to 2.0 µM) was determined using the optimized EIS assay, whereas the limits of detection and quantification were found to be 298 and 994 fM, respectively. Worthy, the fabricated biosensor was tested using mismatched oligos based on the investigated signals of the matched SARS-CoV-2 oligos. The complete mismatched and mismatched in one nucleotide were used and the chips respond selectively towards the matched oligos while they have a 40% impedimetric response in case of the completely mismatched oligos. Furthermore, a 79% response was obtained towards the one-mismatched oligos. Notably, the fabricated biosensor is selective for viral nucleic acid detection.

## Supplementary Information

Supplementary Information.

## Data Availability

All Data are available in the current version of the manuscript. The datasets analyzed during the current study are available from the corresponding author upon reasonable request. All based nucleotide retrieved from GeneBank-NCBI (https://www.ncbi.nlm.nih.gov/genome/browse/#!/viruses/86693).
