# Supplementary material for: Designing and fabrication of electrochemical nano-biosensor for the fast detection of SARS-CoV-2-RNA
Source: Sci Rep. 2023 Mar 29;13:5139. doi: 10.1038/s41598-023-32168-5 (PMC10054215; doi:10.1038/s41598-023-32168-5)
Supplement: Supplementary file 1 — Supplementary Information. [file 41598_2023_32168_MOESM1_ESM.docx]

**Designing and Fabrication of Electrochemical Nano-RNA biosensor for the Fast Detection of SARS-CoV-2**

**Heba A. Hussein^1*^, Amro Hanora^2^, Samar M. Solyman^2^, Rabeay Y. A. Hassan^3*^**

^1^Virology Department, Animal Health Research Institute (AHRI), Agricultural Research Center (ARC), Giza, 12619, Egypt;

^2^Department of Microbiology & Immunology, College of Pharmacy, Suez Canal University, Ismailia, Egypt;

^3^Nanoscience Program, University of Science and Technology (UST), Zewail City of Science and Technology, Giza 12578, Egypt;

***Corresponding authors:**

Rabeay Y. A. Hassan, PhD

Nanoscience Program, University of Science and Technology (UST),

Zewail City of Science and Technology,

6th October City, 12578 Giza, Egypt

Email: [ryounes@zewailcity.edu.eg](mailto:ryounes@zewailcity.edu.eg) or [rabeayy@gmail.com](mailto:rabeayy@gmail.com)

ORCID: <https://orcid.org/0000-0002-1867-9643>

Heba A. Hussein, Ph.D.

Virology Department, Animal Health Research Institute (AHRI),

Agricultural Research Center (ARC), Giza, 12619, Egypt,

Email: [hebaahmed@ahri.gov.eg](mailto:hebaahmed@ahri.gov.eg) or [hebaahmedhussein@gmail.com](mailto:hebaahmedhussein@gmail.com)

**ORCID:** <https://orcid.org/0000-0003-1058-4946>

**Supplementary Data**


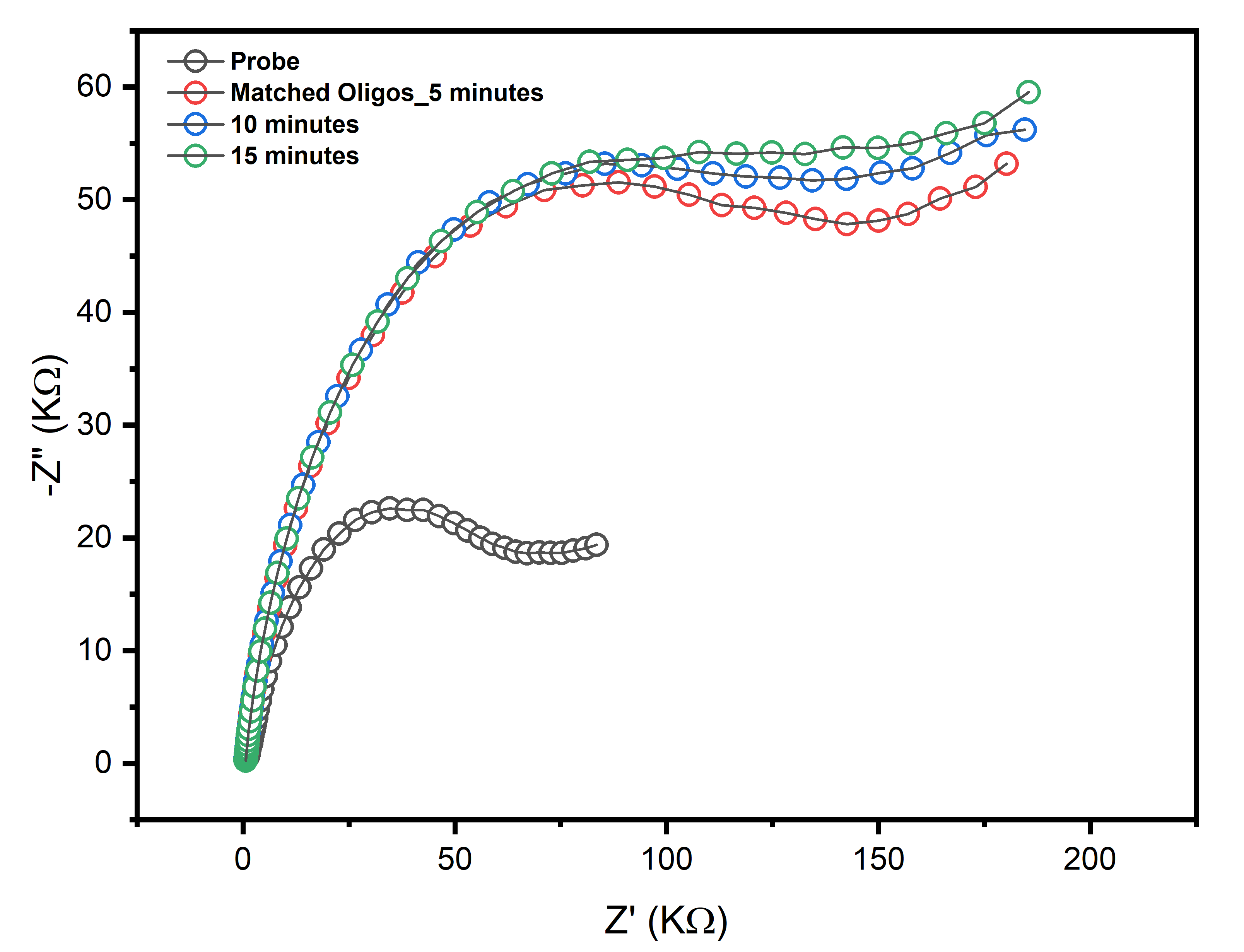


**Figure S1**: Incubation time testing for Target/Probe signature oligos hybridization (ranging from 5 to 15 minutes) at room temperature. 5-10 minutes were enough for the reactant to bind and give the investigated response.
